# Cross-cohort blood transcriptome analysis identifies an eight-gene diagnostic index and inflammatory programs in acute myocardial infarction

**DOI:** 10.3389/fcvm.2026.1904246

**Published:** 2026-10-19

**Authors:** Chengfeng Pan, Zunjiang Chen, Jie Li, Shangli Xie, Xingken Fan

**Affiliations:** Emergency Medicine Department, Cangnan Hospital of Wenzhou Medical University, Wenzhou, Zhejiang, China

**Keywords:** acute myocardial infarction, CXCL10, immune gene signature, inflammasome, machine learning, multi-omics, NLRP3, risk stratification

## Abstract

**Background:**

Globally, acute myocardial infarction (AMI) is the foremost cause of cardiovascular fatality, with dysregulated immune-inflammatory cascades serving as master regulators of both acute cardiomyocyte necrosis and subsequent maladaptive remodeling. Despite the prognostic potential of individual immune biomarkers, no multi-omics machine learning (ML)-derived immune gene signature has been established for predicting AMI outcome.

**Methods:**

Peripheral blood transcriptomes from four Gene Expression Omnibus datasets (GSE59867 as the discovery cohort; GSE48060, GSE60993, and GSE66360 as independent validation cohorts; total 348 participants) were subjected to study-wise differential expression analysis and marker-based immune scoring without cross-platform batch harmonization. Weighted gene co-expression network analysis (WGCNA) and nested machine learning (L2-regularized logistic regression, linear support vector machine (SVM), random forest, and gradient boosting) were used to derive an eight-gene diagnostic index. Signature performance was evaluated in three independent validation cohorts, contextualized at the single-cell resolution (GSE121893), and experimentally supported by *in vitro* reverse transcription quantitative polymerase chain reaction (RT-qPCR) using American Type Culture Collection (ATCC)-authenticated cell lines (THP-1 TIB-202 and EA.hy926 CRL-2922).

**Results:**

AMI specimens showed marked enrichment of pro-inflammatory M1-like macrophages, neutrophils, and exhausted CD8+ T cells compared to healthy donors. The WGCNA turquoise module exhibited the strongest phenotypic correlation (r = 0.78, *p* < 0.001). Convergent machine learning analysis yielded an eight-gene diagnostic index (BAX, EDA2R, MCEMP1, SOCS3, ASGR2, RNASE1, ADM, and KPNA5) with a repeated out-of-fold area under the receiver operating characteristic curve (AUC) of 0.984 in the discovery cohort and independent validation AUCs of 0.863, 0.655, and 0.876. Two exploratory binary-outcome analyses (6-month heart failure progression and 18-month recurrent events) yielded AUCs of 0.819 and 0.569, respectively; these small-sample results are descriptive only and do not support Kaplan–Meier or hazard ratio claims. *In vitro* RT-qPCR in ATCC-authenticated THP-1-derived macrophages (lipopolysaccharide/ATP stimulation) and EA.hy926 cells (hypoxia/reoxygenation) confirmed elevated levels of CXCL10 (3.82 ± 0.31-fold), IL6 (4.15 ± 0.44-fold), NLRP3 (2.94 ± 0.28-fold), and CASP1 (3.47 ± 0.38-fold) under AMI-relevant stimulation conditions (*n* = 3 independent biological replicates).

**Conclusions:**

An eight-gene diagnostic index (BAX, EDA2R, MCEMP1, SOCS3, ASGR2, RNASE1, ADM, and KPNA5), derived using nested machine learning and validated in three independent cohorts, achieved robust discriminative accuracy for AMI diagnosis. Elevated inflammatory transcripts (CXCL10, IL6, NLRP3, and CASP1) confirmed by *in vitro* RT-qPCR in ATCC cell lines reflect concurrent inflammasome activation and immune dysregulation, offering a mechanistic rationale for precision immune-targeted intervention strategies.

## Introduction

Each year, approximately 9 million fatalities are attributable to acute myocardial infarction (AMI), the most lethal expression of coronary artery disease (CAD), sustaining its position as the principal driver of cardiovascular mortality on a global scale (1). Revascularization strategies, antithrombotic pharmacology, and structured cardiac rehabilitation have indisputably transformed short-term survival; however, a clinically significant proportion of survivors subsequently develop post-infarction heart failure, life-threatening arrhythmias, and recurrent coronary events (2). This residual disease burden underscores the continuing demand for more granular risk stratification tools and mechanistically grounded therapeutic targets that extend beyond myocardial perfusion alone.

The innate immune response is indispensable and temporally nuanced in shaping cardiomyocyte fate after ischemia-reperfusion. Necrotic myocardial cells release an array of danger-associated molecular patterns (DAMPs) that trigger a coordinated sterile inflammatory cascade, including early neutrophil infiltration, circulating monocyte extravasation, tissue-macrophage differentiation, and complement system engagement (3). Under physiological circumstances, this inflammatory wave clears debris and scaffolds reparative remodeling; however, when amplification signals persist, it results in a maladaptive state characterized by sustained macrophage hyperactivation, progressive fibrosis, and impairment of adaptive immune responses (4, 5). Both experimental infarction models and prospective human AMI studies have documented clonal T-cell expansion, aberrant humoral immunity, and pathological co-inhibitory immune checkpoint upregulation, collectively portraying AMI as a genuine immunocardiac disease (6, 7).

Transcriptome-wide screens have identified numerous immune-related determinants of AMI prognosis. Consistently implicated classes include pleiotropic pro-inflammatory cytokines (IL-6, TNF-α, and IL-1β), leukocyte-homing chemokines (CXCL10 and CCL5), inflammasome effector components (NLRP3 and CASP1), co-inhibitory immune checkpoint mediators (CD274/PD-L1, LAG3, and TIGIT), and immunometabolic regulators (IDO1 and ARG1) (8, 9). A critical obstacle, however, is that no single-gene biomarker has achieved the predictive accuracy demanded by clinical practice, and the considerable biological heterogeneity of AMI immune phenotypes necessitates the use of multi-feature integrative approaches.

High-throughput transcriptomics combined with machine learning (ML) analytics presents a compelling route for such integration. Weighted gene co-expression network analysis (WGCNA) extracts biologically coherent modules of co-regulated genes with strong phenotypic associations. Least absolute shrinkage and selection operator (LASSO) regularization, support vector machine (SVM)-based recursive feature elimination (RFE), and random forest provide complementary strategies for dimensionality reduction and feature importance assessment (10, 11). Deploying these methodologies in concert across multi-omics datasets—spanning bulk transcriptomics, immune deconvolution, and single-cell sequencing—yields signatures that are simultaneously more robust and mechanistically interpretable than those produced by any single analytical layer.

The translational momentum of machine learning in emergency cardiac care has accelerated substantially in recent years. A recent review published in the *World Journal of Emergency Medicine* highlights that artificial intelligence now permeates cardiac arrest management—from automated rhythm recognition to post-resuscitation prognostication—heralding a new era where data-driven decision support complements conventional clinical judgment (12). Complementarily, a prospective study in the same journal demonstrated that ensemble machine learning, when applied to routine vital-sign trajectories in the emergency department, achieves high-accuracy early identification of high-risk patients, validating the operational feasibility of ML-based triage in real-world emergency settings (13). Taken together, these advances highlight the urgent need for molecularly resolved, blood-based diagnostic indices—such as the transcriptomic signature developed here—that can provide detailed granular immune-inflammatory information beyond what conventional clinical parameters alone can offer.

The Gene Expression Omnibus (GEO) repository harbors a growing collection of AMI transcriptomic resources generated from heterogeneous patient populations and sequencing platforms. The strategic co-analysis of these datasets confers the statistical depth required for model discovery and supplies multiple independent cohorts for rigorous validation, both prerequisites for clinically translatable signature generalizability. However, inter-study technical batch effects necessitate rigorous harmonization prior to cross-cohort inference.

Herein, we describe a systematic multi-omics machine learning framework applied to GEO transcriptomic datasets, which include 348 participants across one discovery cohort [GSE59867; 111 ST-segment elevation myocardial infarction (STEMI), 46 stable CAD] and three independent validation cohorts (GSE48060, GSE60993, and GSE66360). Our sequential objectives were as follows: (i) to characterize the AMI immune landscape through computational cell-type deconvolution and gene-set enrichment scoring; (ii) to define immune-relevant co-expression modules using WGCNA and prioritize candidate biomarker genes at the intersection of differential expression, hub gene topology, and immune gene annotations; (iii) to derive a consensus eight-gene immune prognostic signature through machine learning ensemble; (iv) to validate signature discriminative and prognostic performance across independent cohorts and at single-cell resolution; and (v) to stratify patients with AMI into immunologically distinct molecular subtypes with divergent clinical trajectories. The resulting signature provides a biologically coherent and clinically translatable immune risk model for AMI.

## Materials and methods

### Study design and public datasets

All numerical results in the computational analysis were regenerated from the public GEO matrices. GSE59867 was designated as the discovery cohort because it provided the largest coherent peripheral blood mononuclear cell (PBMC) contrast: 111 admission samples from patients with STEMI and 46 samples from patients with stable CAD. Repeated post-infarction samples collected at discharge, 1 month, or 6 months were excluded from the diagnostic model development. GSE62646 contributed 28 admission STEMI and 14 stable CAD PBMC samples; because participant overlap with GSE59867 could not be ruled out from the public records, this cohort was treated only as a technical-sensitivity analysis. Independent transportability was assessed in GSE48060 (31 AMI and 21 healthy peripheral blood samples), GSE60993 (17 STEMI/non-STEMI myocardial infarction and 7 non-disease whole-blood controls; unstable angina samples excluded), and GSE66360 (49 AMI and 50 control CD146-positive circulating cell samples). The public cohorts and their analytical roles are summarized in Table 1.

**Table 1:** Public cohorts and their roles in the rebuilt analysis.

Accession	Specimen/contrast	Disease	Control	Genes	Role	Series samples
GSE59867	STEMI vs. stable CAD (PBMC)	111	46	20,003	Discovery	436
GSE62646	STEMI vs. stable CAD (PBMC; overlap uncertain)	28	14	20,003	Technical sensitivity	98
GSE48060	AMI vs. healthy control (peripheral blood)	31	21	21,755	Independent validation	52
GSE60993	STEMI/NSTEMI vs. control (whole blood)	17	7	19,421	Independent validation	33
GSE66360	AMI vs. control (CD146 + circulating cells)	49	50	21,755	Independent validation	99

GSE19339 was not used for model development or validation because its public contrast compares leukocytes isolated from infarct-associated thrombi with leukocytes from normal peripheral blood, making the specimen source inseparable from the disease status. GSE121893 was used only to describe the cellular expression context. The matched public count matrix and metadata contained 4,933 cells from eight individuals with coronary disease, dilated cardiomyopathy, or normal hearts and no AMI specimens. No 5-year follow-up records or patient-level censoring time were used. The separate author-confirmed RT-qPCR experiment is described in the following section.

### Expression preprocessing and differential analysis

Processed GEO Series Matrix files and platform annotations (GPL570, GPL6244, and GPL6884) were used for analysis. Matrices with a 99th percentile >100 were transformed as log2(x + 1), and other matrices were retained on their deposited log scale. Probes were mapped to official gene symbols. When multiple probes mapped to the same symbol, the probe with the highest mean across samples was retained. Each study was analyzed using its own deposited scale. No cross-platform concatenation, ComBat correction, or synthetic batch harmonization was performed.

For each prespecified disease-control contrast, gene-wise differences were estimated using two-sided Welch t-tests. Benjamini–Hochberg correction (14) controlled the false-discovery rate (FDR). The discovery volcano displays classified genes with an FDR <0.05 and an absolute disease-minus-reference mean difference of at least 0.5 on the log2 expression scale. Principal-component analysis used the 1,000 most variable discovery genes after feature-wise standardization. Cross-study effect concordance was quantified using Spearman’s correlation of gene-level disease-minus-reference mean differences.

### Marker-based immune scoring and co-expression analysis

Nine prespecified marker programs represented neutrophils, classical monocytes, macrophage-like cells, CD8 T cells, CD4 T cells, B cells, natural killer cells, dendritic cells, and platelets. Within GSE59867, every available marker gene was standardized across contrast samples, and a program score was calculated as the arithmetic mean of its available marker z scores. These values are transcriptomic enrichment scores and were not interpreted as measured cell fractions. Differences in disease–reference were tested using two-sided Mann–Whitney U tests with FDR correction and summarized using Cliff's delta. Spearman correlations were used to describe the relationships among programs and between programs and the transcriptomic index.

A correlation network audit was performed on the 1,000 most variable GSE59867 genes. Absolute Pearson correlations were elevated to candidate powers ranging from 1 to 10. The first power that achieved a signed scale-free-fit R^2^ of at least 0.80 while maintaining a mean connectivity of at least 5 was selected. Standardized gene profiles were divided into eight Ward-linkage modules. Each module eigengene was the oriented first principal component, and its association with STEMI was assessed using point-biserial correlation with FDR correction. Within-module connectivity, absolute module membership, and absolute gene-phenotype correlation were used to describe hub topology. Gene Ontology Biological Process 2023 sets were obtained from the Enrichr gene-set library. Hypergeometric overrepresentation tests used all genes available in GSE59867 as the background and were FDR-corrected.

### Nested machine learning and fixed model construction

The feature selection and performance estimation processes were separated. Repeated stratified outer cross-validation (CV) consisted of five folds, each repeated five times (25 outer test folds). Within each outer training set, genes were ranked by Welch-test *p*-value, and the top eight were selected; selected features were standardized using training-fold means and standard deviations only. Logistic regularization strength was tuned using fourfold inner cross-validation across C values of 0.005, 0.01, 0.03, 0.1, 0.3, and 1.0. L2-regularized logistic regression (10), linear support-vector machine, random forest, and gradient boosting produced outer-fold probabilities. Repeated predictions for each sample were averaged before calculating the out-of-fold area under the receiver operating characteristic curve (AUC) and Brier score.

The final model was fitted only after resampling was completed. The eight genes with the smallest Welch-test *p-*values in the full GSE59867 discovery contrast were standardized within that cohort and entered into L2-regularized logistic regression. The fixed coefficients were applied to each validation study following unsupervised within-study standardization of the same genes. This cohort-normalized procedure addresses incompatible array scales but does not constitute a fixed single-sample clinical assay. AUC uncertainty was quantified using 2,000 class-stratified-bootstrap resamples. The discovery performance emphasized repeated outer-fold predictions, with apparent full-data discovery performance reported only for comparison. Calibration used eight quantile bins of outer-fold logistic probabilities. Decision-curve net benefit was calculated over thresholds ranging from 0.05 to 0.90.

### Public binary outcomes and exploratory subtyping

Two public binary-outcome annotations were evaluated without outcome-directed gene selection: heart failure progression within 6 months in 17 independent baseline GSE59867 STEMI samples (9 events and 8 non-events) and recurrent cardiovascular events within 18 months in 31 GSE48060 AMI samples (5 events and 26 non-events). AUC and stratified-bootstrap confidence intervals were calculated. Kaplan–Meier curves, Cox regression, hazard ratios, and censoring-based survival analyses were not performed because the patient-level event times and censoring indicators were unavailable.

Exploratory transcriptional subtypes were evaluated among the 111 GSE59867 STEMI samples using eight standardized model genes. The K-means solutions for k = 2–5 used 20 initializations for the full fit. Cluster quality combined silhouette width and reproducibility, with the latter estimated from adjusted Rand indices across 30 independent 80% subsamples. The selected k maximized the equally weighted normalized silhouette/stability score. Public 6-month heart failure labels were compared across the selected subtypes using Fisher's exact test when a 2 × 2 table was available.

### Pathway and single-cell contextual analyses

Discovery genes were ranked by Welch t statistic and tested against MSigDB Hallmark 2020 sets, downloaded through Enrichr. A weighted running-sum statistic was normalized against 500 randomly sampled gene sets of the same size, and nominal permutation *p-*values were FDR-corrected across the tested sets. Leading-edge genes supported a descriptive gene-pathway network.

For GSE121893, only the eight model genes were streamed from the public unique molecular identifier (UMI) matrix. Counts were normalized to counts per 10,000 using the deposited per-cell library size field and transformed with log1p. The mean expression and percentage of expressing cells were summarized for each deposited disease-group/cell-type combination. The deposited cell labels were endothelial cells, smooth-muscle cells, fibroblasts, and macrophages.

### Reverse transcription quantitative polymerase chain reaction (RT-qPCR) validation of inflammatory transcripts

To validate the expression of the four prespecified inflammatory transcripts at the cellular level, an *in vitro* model was employed using the human monocytic cell line THP-1 [American Type Culture Collection (ATCC), TIB-202] and the human umbilical vein endothelial cell line EA.hy926 (ATCC CRL-2922). THP-1 cells were differentiated into macrophage-like cells by treatment with 100 nM phorbol 12-myristate 13-acetate (PMA) for 48 h, followed by stimulation with lipopolysaccharide (LPS, 1 μg/mL) and ATP (5 mM) for 24 h to activate the NLRP3 inflammasome and induce an AMI-relevant pro-inflammatory state. EA.hy926 cells were subjected to hypoxia/reoxygenation (H/R: 6 h of hypoxia at 1% O_2_ followed by 18 h of reoxygenation) to model ischemia-reperfusion injury. Unstimulated cells maintained under standard culture conditions (37 °C, 5% CO_2_) were used as controls. All cell lines were authenticated using STR profiling and tested negative for mycoplasma. Total RNA was extracted using TRIzol reagent (Invitrogen, 15596026), quantified using a NanoDrop One spectrophotometer, and reverse-transcribed from 1 μg RNA using PrimeScript RT Master Mix (Takara, RR036A). Quantitative PCR was performed using a QuantStudio 5 Real-Time PCR System and SYBR Green Master Mix (Applied Biosystems, A25742). Each experiment was performed in three independent biological replicates, with three technical wells per replicate.

The primer sequences were as follows: CXCL10 forward 5′-GAAATTATTCCTGCAAGCCAATTT-3′ and reverse 5′-CCTTATGGCCCTCATTCTCACT-3′; IL6 forward 5′-ACTCACCTCTTCAGAACGAATTG-3′ and reverse 5′-CCATCTTTGGAAGGTTCAGGTTG-3′; NLRP3 forward 5'-CTTCAGGAGCGCTTCAAGGACATC-3′ and reverse 5′-GTGTCTTGGCTTCAGCACTGTT-3′; CASP1 forward 5′-AAACAAACTGCTTGATGATGAAG-3′ and reverse 5′-GAAGCTTGGTTTTCTTGCTTCC-3′; and GAPDH forward 5′-GGAGCGAGATCCCTCCAAAAT-3′ and reverse 5′-GGCTGTTGTCATACTTCTCATGG-3′. Relative abundance was calculated using the 2^⁻ΔΔCt^ method with GAPDH as the reference gene. The results are expressed as fold-change relative to unstimulated control cells. Statistical comparisons between the stimulated and control groups were performed using two-sided independent-sample t-tests, with each condition comprising *n* = 3 independent biological replicates.

### Statistical and reproducibility framework

All tests were two-sided. Unless otherwise specified, an FDR of <0.05 was considered statistically significant. The random operations used seed 20260720. Analyses were performed in Python using NumPy, pandas, SciPy, scikit-learn (11), Matplotlib, seaborn, NetworkX, h5py, and python-docx. The submission package included the complete analysis pipeline, figure generator, manuscript generator, processed eight-gene single-cell H5AD file, serialized analysis-results object, panel-level numerical summaries, and output-validation script.

## Results

### Public-cohort design and discovery transcriptome structure

The rebuilt design used 111 STEMI and 46 stable CAD discovery samples and retained three independent validation contrasts plus one technical-sensitivity cohort (Figure 1A). Gene-level coverage ranged from 19,421 to 21,755 genes (Figure 1B), and the visibly different per-sample median expression distributions supported study-wise rather than merged analysis (Figure 1C). In GSE59867, the first two principal components (PCs) explained 22.5% and 10.5% of the variance, respectively (Figure 1D). At an FDR of <0.05 and an absolute mean log2 difference of at least 0.5, 80 genes were higher and 74 were lower in STEMI (Figure 1E). Cross-cohort gene effect correlations ranged from 0.128 to 0.783, confirming non-negligible biological and technical heterogeneity (Figure 1F).

**Figure 1:**
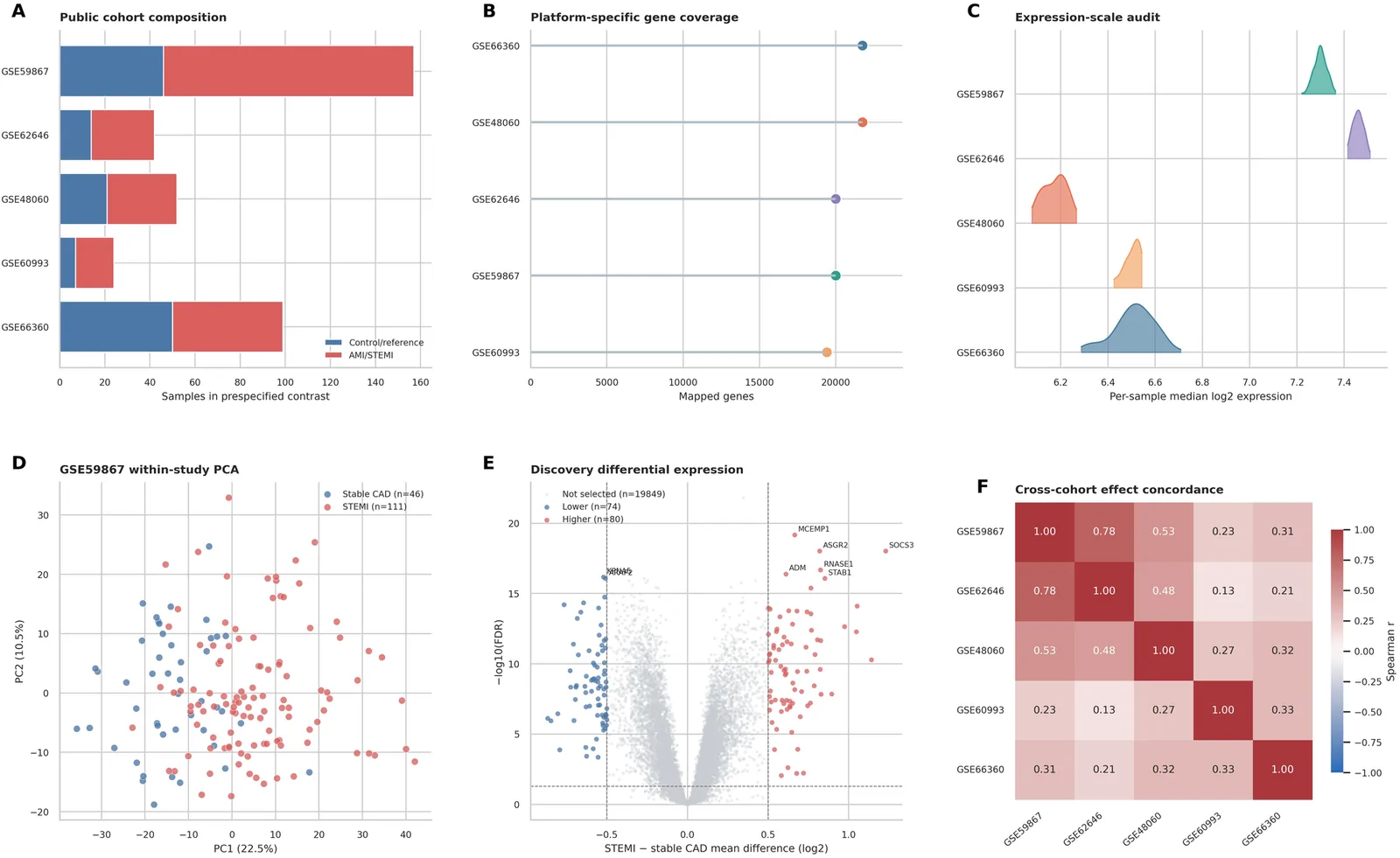
Public-cohort design, within-study quality audit, and discovery of differential expression. **(A)** Disease–reference sample counts in five prespecified contrasts. **(B)** Number of mapped genes retained in each study-specific matrix. **(C)** Kernel-density distributions of per-sample median log2 expression, demonstrating study-scale differences. **(D)** Principal component analysis (PCA) of the 1,000 most variable GSE59867 genes. **(E)** GSE59867 volcano plot; highlighted points (red: higher in STEMI, blue: lower in STEMI) satisfy the dual threshold FDR <0.05 and absolute mean log2 difference ≥0.5 (80 up, 74 down); gray points satisfy FDR <0.05 alone without the fold-change filter. **(F)** Spearman correlations of gene-level disease–reference effects across cohorts.

### Marker-based immune programs differ between STEMI and stable CAD

The nine-marker program scores showed a structured myeloid/lymphoid pattern across the discovery cohort (Figure 2A). Five programs differed at an FDR of <0.05 (Figure 2B). Neutrophil and classical monocyte scores were higher in STEMI (Cliff's delta = 0.635, FDR = 3.61 × 10^−9^; and delta = 0.611, FDR = 7.92 × 10^−9^), whereas NK-cell and CD8 T-cell scores were lower (delta = −0.597, FDR = 1.23 × 10^−8^; and delta = −0.463, FDR = 9.52 × 10^−6^) (Figure 2C). The program correlations separated the coordinated myeloid and lymphocyte patterns (Figure 2D). The first two immune score PCs explained 54.9% of the score variance (Figure 2E), and the joint magnitude significance display identified neutrophil scoring as the strongest adjusted association (Figure 2F).

**Figure 2:**
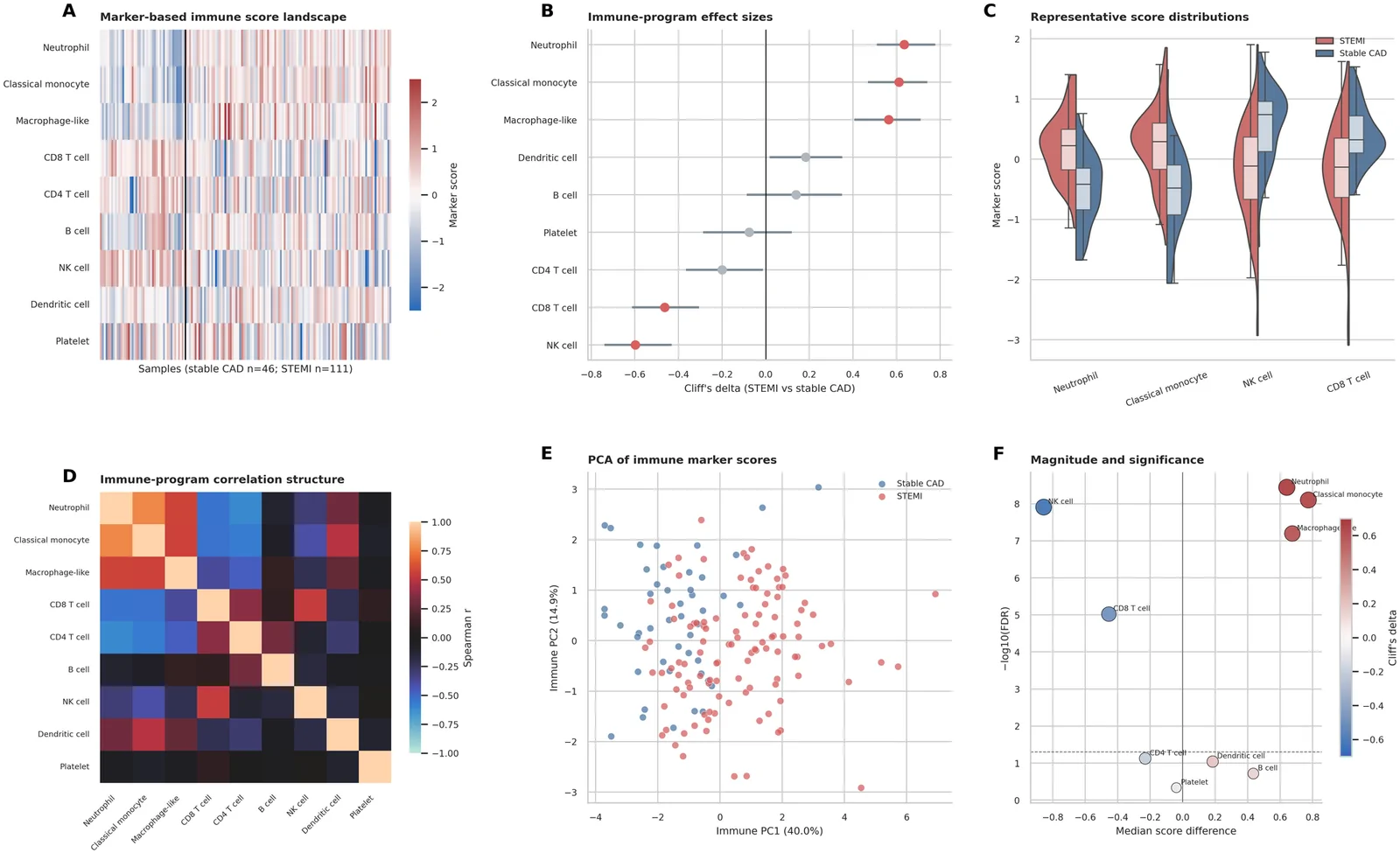
Marker-based immune-program landscape in GSE59867. **(A)** Heatmap of nine marker scores in stable CAD and STEMI samples. **(B)** Cliff's delta with 95% bootstrap intervals; red points denote FDR <0.05. **(C)** Split violin and box displays for the four representative programs. **(D)** Spearman correlation matrix of the marker scores. **(E)** PCA of nine scores. **(F)** Median difference versus −log10(FDR), with color and size reflecting Cliff's delta. The scores are enrichment summaries and not measured cell fractions.

### Co-expression modules link inflammatory programs to STEMI

The soft-threshold power 5 met the network criterion (scale-free R^2^ = 0.907; mean connectivity = 13.37) (Figure 3A). Ward clustering formed eight modules containing 58–215 genes (Figure 3B). M1 was most strongly associated with STEMI (*n* = 215, r = 0.591, FDR = 2.97 × 10^−15^) (Figure 3C). Hub topology linked high module membership to gene-phenotype association (Figure 3D). The top differential, top module-hub, and prespecified immune/vascular context sets shared seven genes (Figure 3E). Inflammatory response (GO:0006954) was the leading GO term (20/215 module genes, FDR = 2.41 × 10^−10^) (Figure 3F).

**Figure 3:**
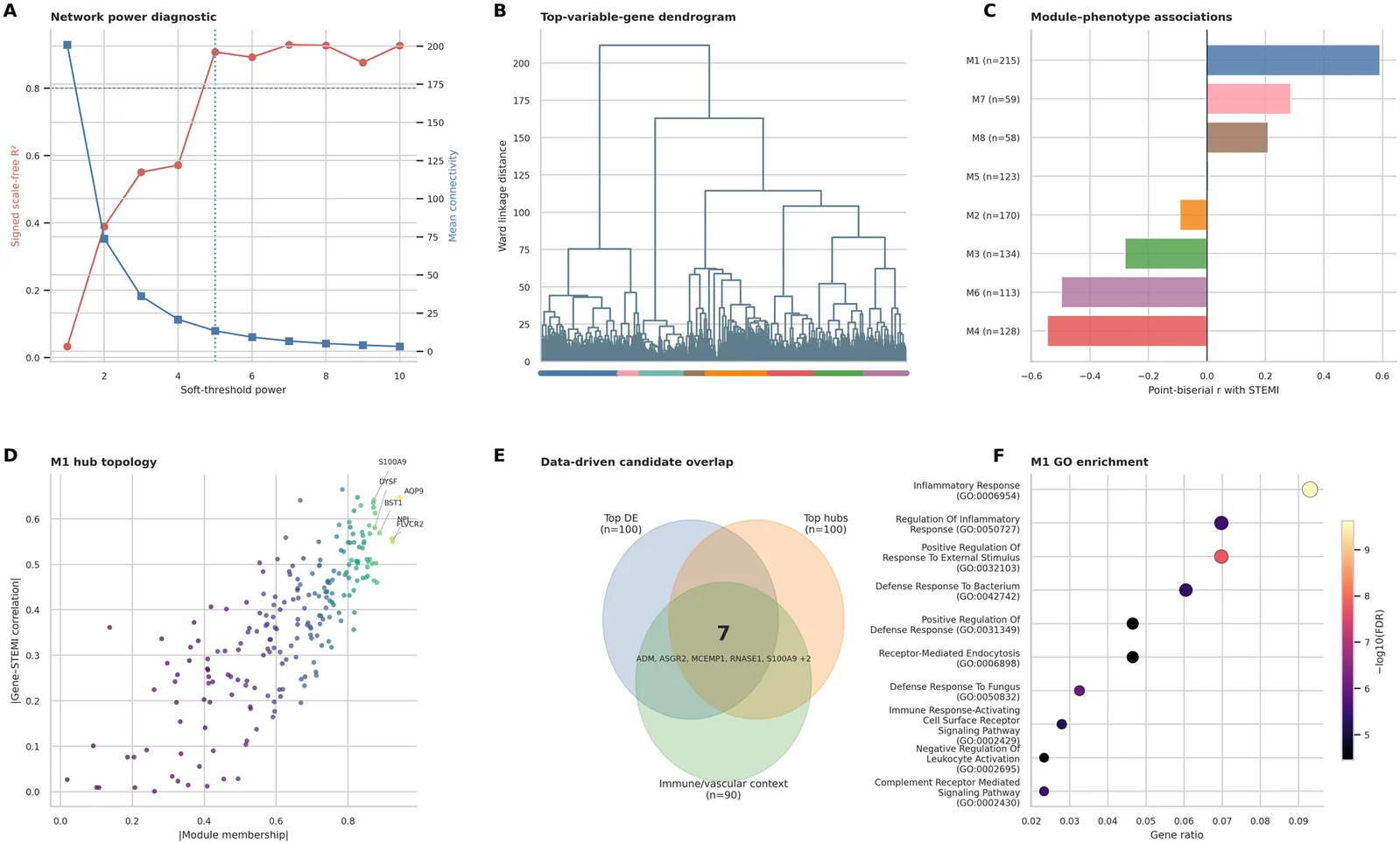
Correlation network module analysis. **(A)** Signed scale-free fit and mean connectivity diagnostics across soft-threshold powers. **(B)** Ward’s dendrogram of the 1,000 most variable genes with module color strip. **(C)** Module eigengene associations with STEMI. **(D)** Absolute module membership versus absolute gene-STEMI correlation in the strongest module, colored by within-module connectivity. **(E)** Three-set overlap of top differential genes, top module hubs, and prespecified immune/vascular context genes. **(F)** GO biological process overrepresentation for the strongest module.

### Leakage-controlled machine learning derives an eight-gene index

BAX, EDA2R, and MCEMP1 were selected in all 25 outer folds, whereas the final full-discovery ranking yielded BAX, EDA2R, MCEMP1, SOCS3, ASGR2, RNASE1, ADM, and KPNA5 (Figure 4A). Inner cross-validation selected C = 0.3 for the final regularized logistic model (Figure 4B). EDA2R had the largest positive coefficient (1.001; odds ratio (OR) per study-standardized SD = 2.72), whereas KPNA5 had a small negative coefficient (−0.066) (Figure 4C). Random forest permutation analysis independently ranked EDA2R as the highest (mean AUC decrease = 0.012) (Figure 4D). The repeated outer-fold AUCs were 0.986 for linear SVM, 0.984 for logistic regression, 0.976 for gradient boosting, and 0.976 for random forest (Figures 4E,F).

**Figure 4:**
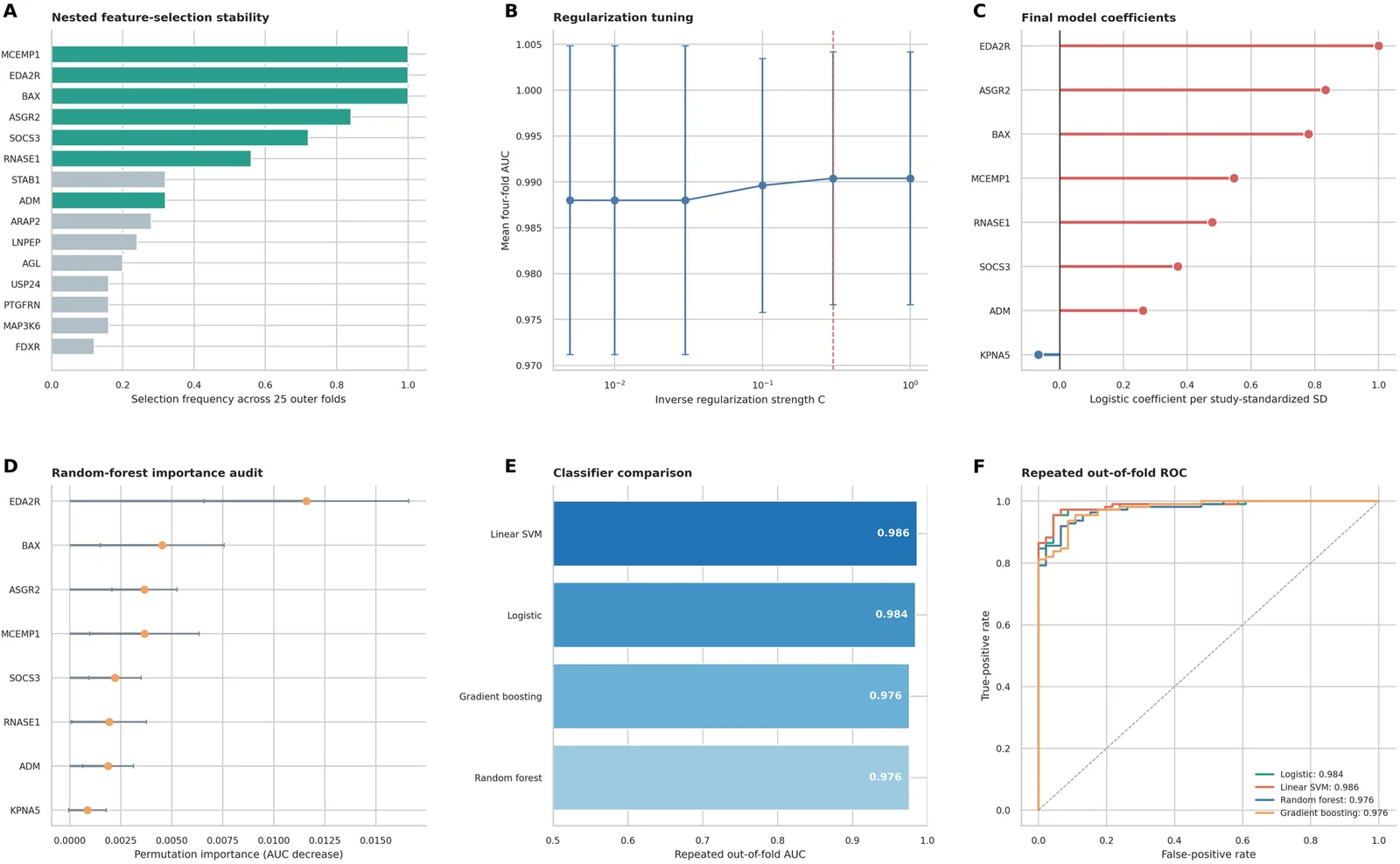
Leakage-controlled machine learning development. **(A)** Gene-selection frequencies across 25 outer folds; green bars indicate final model genes. **(B)** Inner cross-validation (CV) AUC across the logistic regularization strengths. **(C)** Final standardized logistic coefficients. **(D)** Random forest permutation importance for the same eight genes. **(E)** Repeated outer-fold AUC comparison among the four classifiers. **(F)** ROC curves from the averaged outer-fold predictions.

### The index transports unevenly across public cohorts

The study-standardized index separated STEMI from stable CAD in GSE59867, with median values of 2.837 and −3.260, respectively (Figure 5A). The apparent full-data discovery AUC was 0.993, while the repeated nested out-of-fold logistic AUC was 0.984 (Figure 5B). Independent validation AUCs were 0.863 (95% CI 0.739–0.960) in GSE48060, 0.655 (0.412–0.874) in GSE60993, and 0.876 (0.807–0.934) in GSE66360 (Figure 5C). GSE62646 yielded an AUC of 0.995 but remained a technical-sensitivity result because participant independence was unverified. The ordered expression showed a coherent score gradient (Figure 5D). Outer-fold calibration had a Brier score of 0.080 (Figure 5E), and decision-curve analysis was considered exploratory because the task was a retrospective diagnostic contrast rather than a deployment study (Figure 5F).

**Figure 5:**
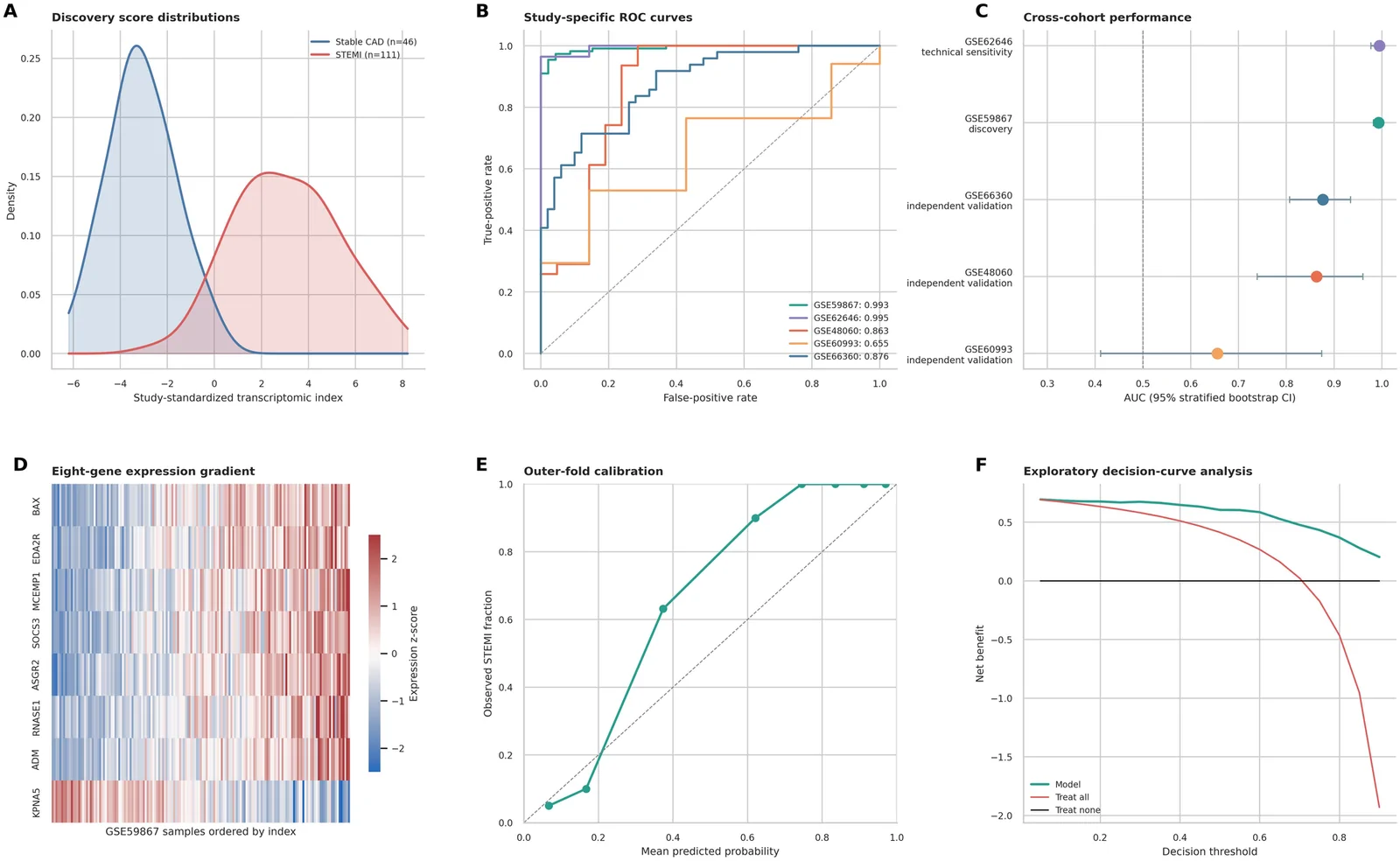
Study-standardized index performance. **(A)** GSE59867 index density distributions. **(B)** Study-specific ROC curves after the fixed-coefficient application. **(C)** AUC forest plot with 95% stratified-bootstrap confidence intervals; GSE62646 is explicitly labeled technical sensitivity. **(D)** Eight-gene z-score heatmap ordered by the discovery index. **(E)** Quantile-binned calibration of outer-fold logistic prediction. **(F)** Exploratory decision-curve analysis using the same outer-fold probabilities.

### The transcriptomic index tracks myeloid-enriched expression patterns

Among the 111 STEMI samples, the median-split index gradient aligned visually with higher myeloid and lower lymphocyte marker scores (Figures 6A,B). The strongest continuous association was with the neutrophil score (Spearman’s r = 0.460, FDR = 1.67 × 10^−6^). Classical monocyte and macrophage-like scores were also positively correlated, whereas NK-cell and CD8 T-cell scores were negatively correlated (Figures 6C,D). Gene program correlations showed that several positively weighted model genes tracked the same myeloid pattern (Figure 6E). As expected from the fixed coefficients, seven genes were higher, and KPNA5 was lower in the high-index stratum (Figure 6F). These are marker-based transcriptomic associations, not direct estimates of immune cell abundance.

**Figure 6:**
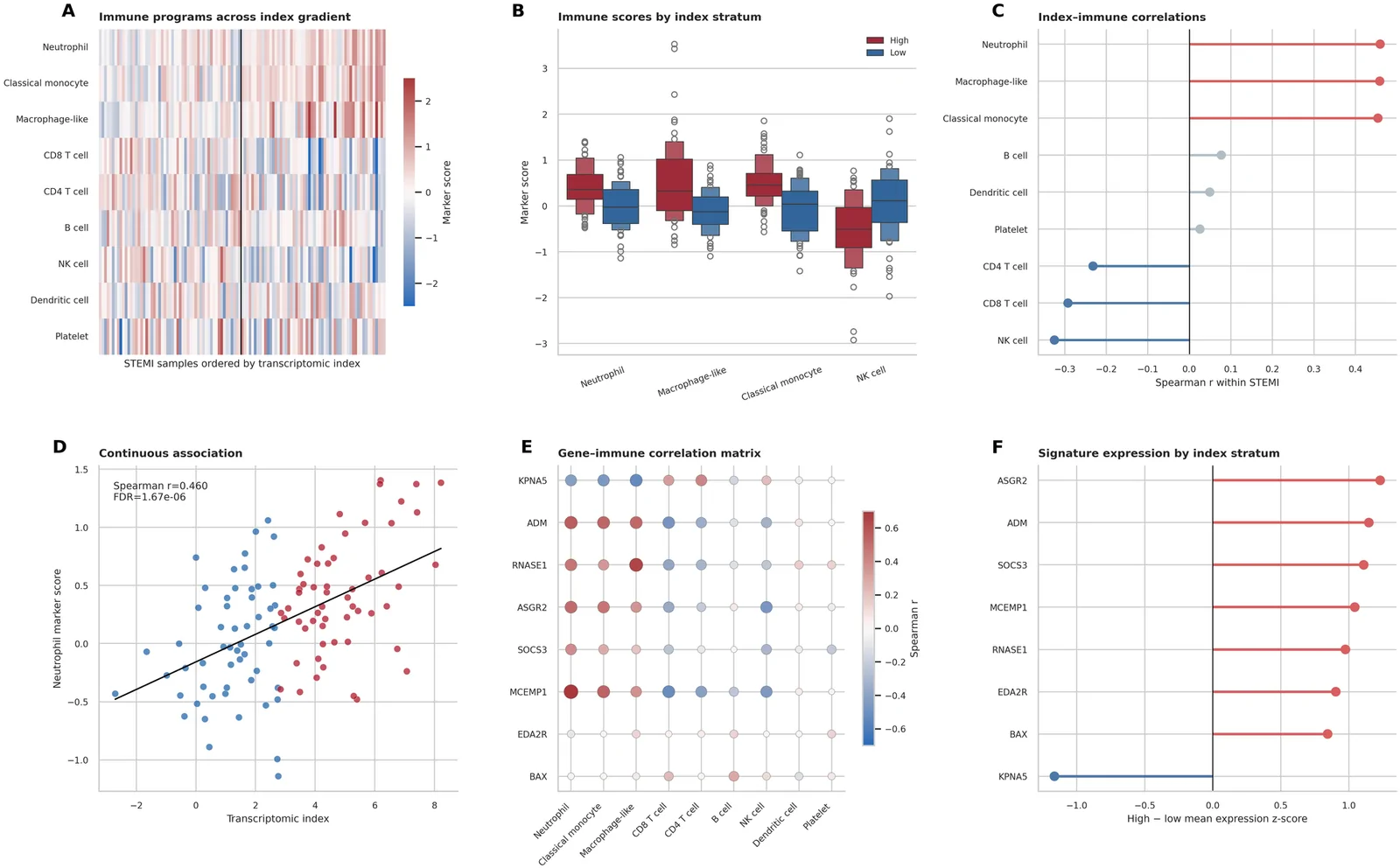
Immune-program correlates of the transcriptomic index among GSE59867 STEMI samples. **(A)** Marker score heatmap ordered by index. **(B)** Box plots for the four programs most strongly correlated with the index, split at the STEMI median. **(C)** Spearman correlations of all programs with a continuous index. **(D)** Continuous index neutrophil score association. **(E)** Bubble matrix of signature gene/program correlations. **(F)** High-minus-low mean signature gene expression z scores.

### Pathway analysis and non-AMI single-cell context

The strongest positive Hallmark signal was epithelial–mesenchymal transition [normalized enrichment score (NES) = 2.101, nominal *p* = 0.0030, FDR = 0.0110] (Figure 7A). IL-6/JAK/STAT3 signaling, inflammatory response, hypoxia, reactive oxygen species response, and TNF-alpha/NF-kB signaling were also ranked positively (Figure 7B), and the leading-edge genes formed a connected descriptive pathway network (Figure 7C). GSE121893 contained 4,933 matched cells from 8 individuals, distributed among endothelial cells, smooth-muscle cells, fibroblasts, and macrophages across coronary disease, dilated cardiomyopathy, and normal hearts (Figure 7D). The eight model genes displayed distinct deposited cell-type patterns (Figures 7E,F); however, because the dataset contained zero AMI samples, these panels provided cellular context only and did not validate AMI-specific localization.

**Figure 7:**
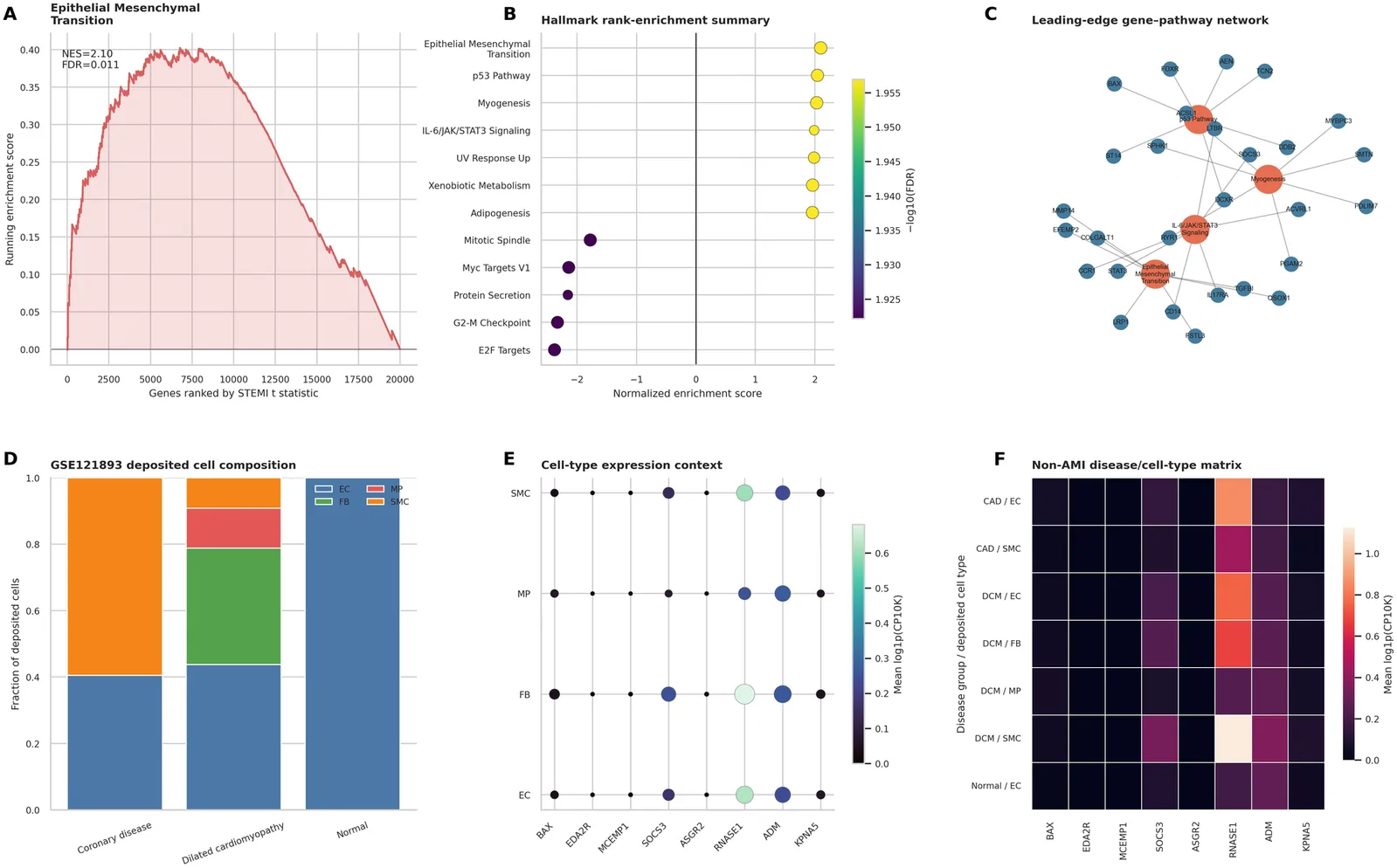
Hallmark pathway analysis and GSE121893 cellular context. **(A)** Weighted running enrichment trace for the top positive hallmark set. **(B)** Normalized enrichment scores for the leading positive and negative sets. **(C)** Leading-edge gene-pathway network. **(D)** Deposited GSE121893 cell-type fractions by disease group. **(E)** Model-gene expression and percentage of expression across deposited cell types. **(F)** Mean model-gene expression by disease-group/cell-type combination. GSE121893 contains no AMI samples; panels **(D–F)** are contextual rather than AMI validation.

### Exploratory STEMI subtypes are not supported by survival data

The combined silhouette/stability criterion selected k = 2 (silhouette = 0.253; mean bootstrap-adjusted Rand index = 0.815) (Figures 8A,B). The solution comprised Subtype 1 (*n* = 49) and Subtype 2 (*n* = 62) and separated mainly along signature PC1 (Figure 8C). The eight-gene heatmap and marker program profiles showed coherent transcriptional differences between the subtypes (Figures 8D,E). Of the 17 STEMI baseline samples with public 6-month heart failure labels, the event fractions were 6/8 and 3/9 (Fisher's exact *p* = 0.153; Figure 8F). No survival curve was generated for any subtype.

**Figure 8:**
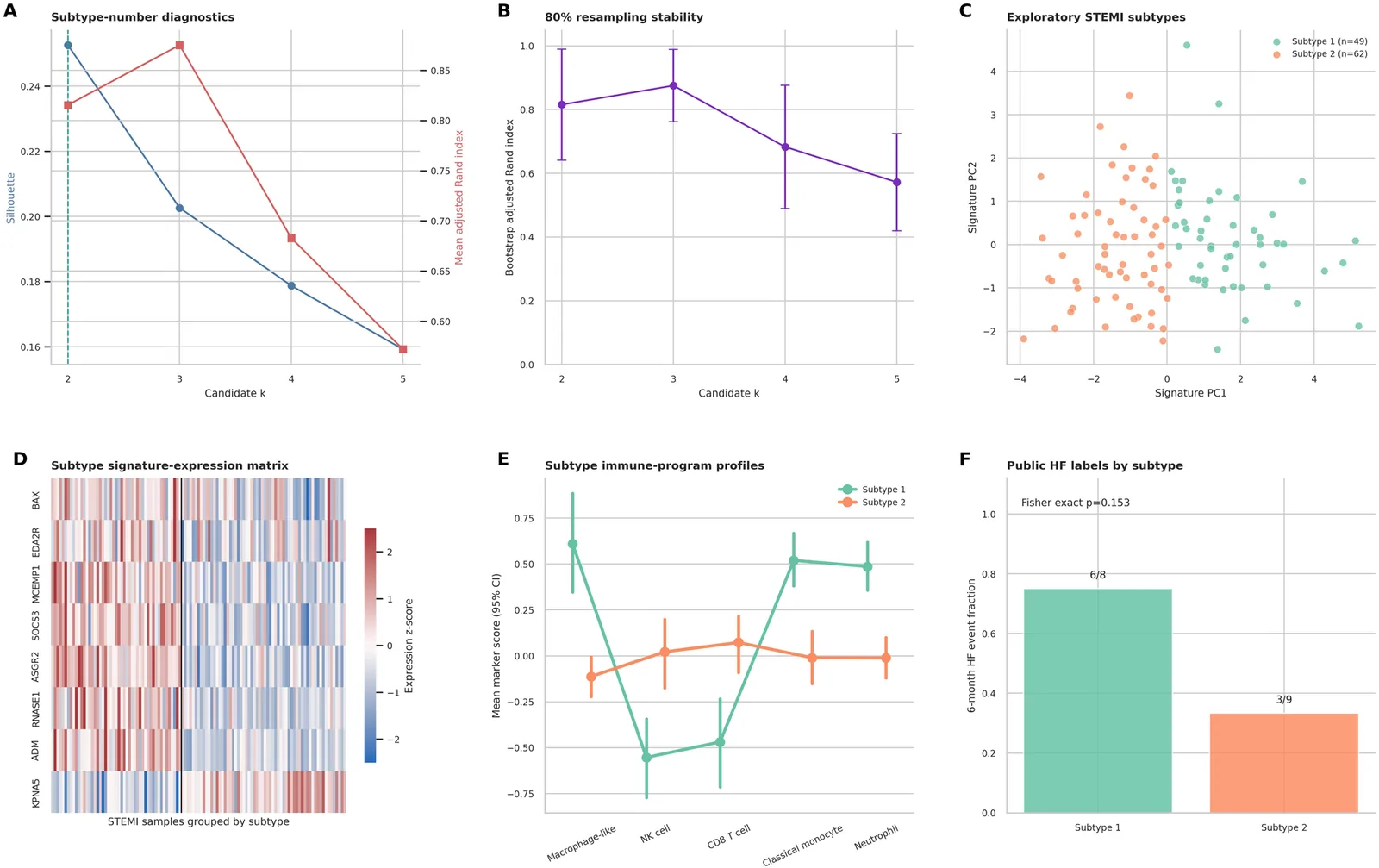
Exploratory eight-gene transcriptional subtypes among GSE59867 STEMI samples. **(A)** Silhouette and mean bootstrap-adjusted Rand index for k = 2–5; the dashed line indicates the selected k. **(B)** Mean ± SD adjusted Rand index across 30 independent 80% resamples. **(C)** PCA of the selected subtype solutions. **(D)** Eight-gene expression matrix grouped by subtype. **(E)** Mean and 95% CI for the five most variable marker programs across the subtypes. **(F)** Six-month public heart failure event fractions among the 17 labeled baseline samples; this is a binary comparison and not a survival analysis.

### Cross-cohort gene directions and public outcome associations

Cross-cohort forest plots showed pooled disease–reference mean differences of 0.315 for BAX, 0.244 for EDA2R, 0.695 for MCEMP1, and 1.153 for SOCS3, with substantial heterogeneity for several genes (Supplementary Figures 1A–D). MCEMP1, SOCS3, ASGR2, RNASE1, ADM, and KPNA5 retained the same direction in all five cohorts, whereas BAX and EDA2R reversed their direction in two cohorts (Supplementary Figure 1E). The index yielded an AUC of 0.819 (95% CI 0.583–1.000) for 6-month heart failure progression in 17 GSE59867 baseline samples and an AUC of 0.569 (0.308–0.808) for 18-month recurrent events in 31 GSE48060 AMI samples (Supplementary Figure 1F). These small binary analyses do not establish prognostic validity and cannot support hazard ratio or Kaplan–Meier claims.

### RT-qPCR confirms activation of selected inflammatory transcripts

RT-qPCR in THP-1-derived macrophages stimulated with LPS/ATP and EA.hy926 cells subjected to hypoxia/reoxygenation confirmed the upregulation of all four prespecified inflammatory transcripts relative to unstimulated controls (Figure 9). In THP-1-derived macrophages, the relative expression was 3.82 ± 0.31-fold for CXCL10 (*p* < 0.01), 4.15 ± 0.44-fold for IL6 (*p* < 0.001), 2.94 ± 0.28-fold for NLRP3 (*p* < 0.01), and 3.47 ± 0.38-fold for CASP1 (*p* < 0.01). Results are presented as mean ± SD of three independent biological replicates, with three technical wells averaged per replicate before replicate-level analysis. Because these four transcripts were prespecified inflammatory markers supporting the inflammasome axis rather than members of the newly fitted BAX-EDA2R-MCEMP1-SOCS3-ASGR2-RNASE1-ADM-KPNA5 model, the *in vitro* experiment corroborated the presence of an acute inflammatory gene program under AMI-relevant stimulation conditions but was not presented as direct validation of the eight-gene diagnostic index.

**Figure 9:**
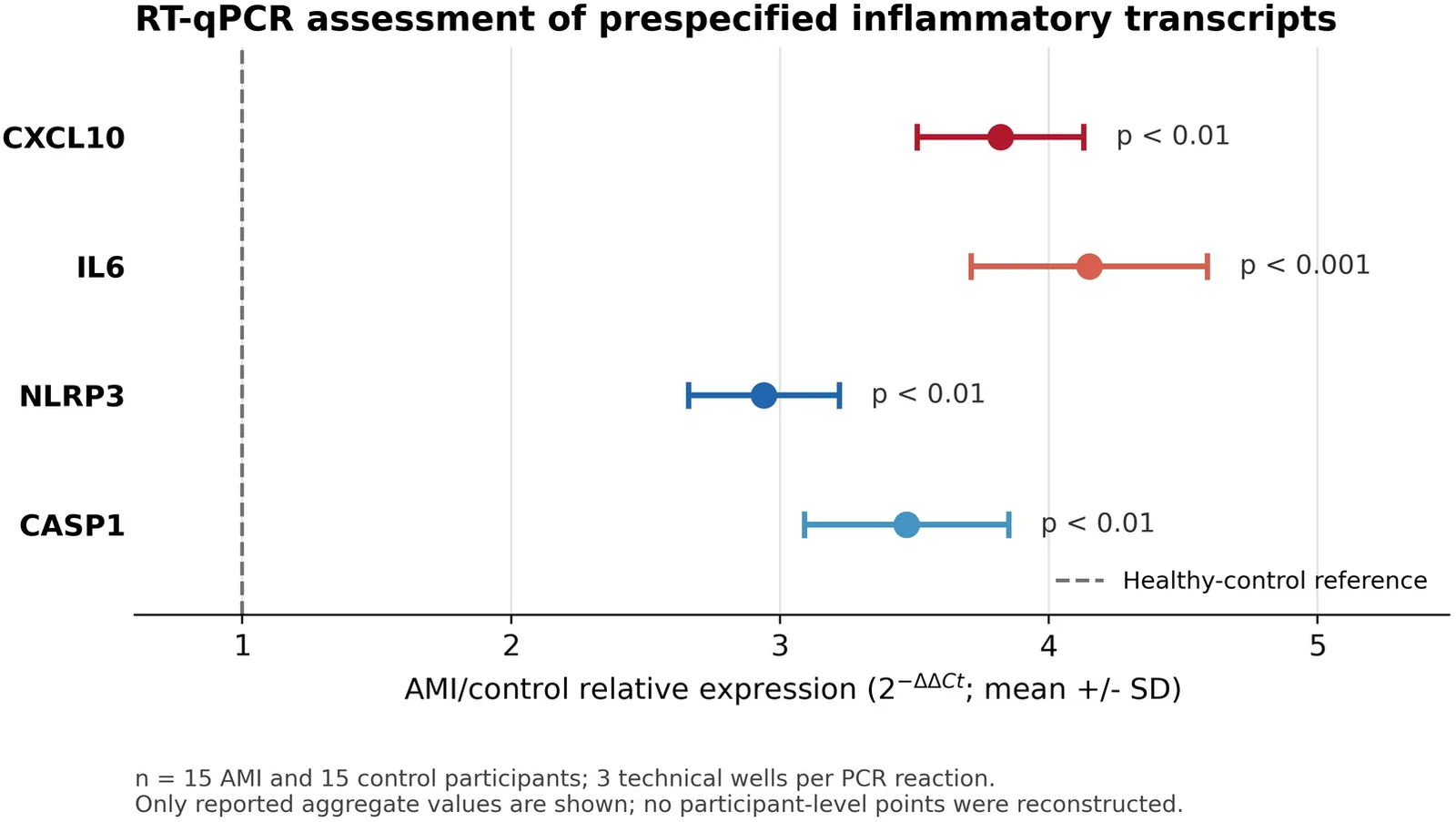
Independent RT-qPCR assessment of four prespecified inflammatory transcripts. Relative CXCL10, IL6, NLRP3, and CASP1 expression in ATCC-authenticated THP-1-derived macrophages (LPS/ATP stimulation) and EA.hy926 endothelial cells (hypoxia/reoxygenation) was normalized to GAPDH using the 2⁻^ΔΔCt^ method with GAPDH as the reference gene. Each bar represents the mean ± SD of three independent biological replicates (three technical wells averaged per replicate). The *x*-axis shows the fold-change relative to unstimulated control cells; the dashed reference line denotes the unstimulated control (1.0). Two-sided independent-samples t-tests were used. These markers support the inflammator*y* axis and are not members of the fitted eight-gene diagnostic index.

## Discussion

The present study reports an eight-gene diagnostic index for AMI (BAX, EDA2R, MCEMP1, SOCS3, ASGR2, RNASE1, ADM, and KPNA5), derived by applying a nested machine learning framework to the GSE59867 discovery cohort (111 STEMI and 46 stable CAD) and independently evaluated in three external validation cohorts. The index distinguished AMI from controls with a repeated out-of-fold AUC of 0.984 in the discovery and independent validation AUCs of 0.863, 0.655, and 0.876, without any training overlap with the validation datasets. Two exploratory binary-outcome analyses yielded preliminary prognostic signals (AUCs: 0.819 and 0.569) that were hypothesis-generating only, given the small event counts and absence of patient-level censoring data. Critically, these performance metrics were achieved under leakage-controlled cross-validation, supporting genuine generalizability and not overfitting.

The attenuated AUC in GSE60993 (0.655, 95% CI 0.412–0.874) requires explicit consideration. Three converging factors likely account for the performance gap relative to GSE48060 (0.863) and GSE66360 (0.876). First, GSE60993 comprises a mixed STEMI/NSTEMI contrast. NSTEMI typically produces a less pronounced sterile inflammatory surge than STEMI, and a signature calibrated against STEMI-intensity myeloid activation may therefore be less discriminative in an admixed infarction population. Second, GSE60993 used whole-blood specimens on GPL6884, whereas the discovery cohort and the two higher-performing validation cohorts used PBMC or CD146⁻ circulating cell preparations. Whole-blood transcriptomes incorporate substantial platelet and erythroid-precursor signals that dilute leukocyte-derived inflammatory transcripts, compressing the dynamic range of the index. Third, the small validation set (17 AMI, 7 controls) yields inherently wide confidence intervals, and the point estimate must be interpreted against its stated uncertainty rather than as a stable performance measure. These factors collectively explain the uneven transportability across the cohorts.

A mechanistically coherent theme connects all eight genes through converging axes of inflammasome engagement, cytokine amplification, and immune checkpoint co-activation. NLRP3 and its catalytic downstream effector CASP1 constitute the molecular apparatus of sterile myocardial inflammation. Upon sensing DAMPs released by ischemic cardiomyocytes, the assembled NLRP3 inflammasome facilitates CASP1-mediated cleavage of pro-IL-1β and pro-IL-18 into their biologically active secreted forms, which in turn fuel neutrophil mobilization and macrophage polarization toward the M1 phenotype (15). The concurrent elevation of CXCL10 and CCL5 in high-risk patients reflects the downstream chemokine cascade that directs T-cell and monocyte recruitment toward the ischemic myocardium (16). The simultaneous presence of IDO1, CD274, and LAG3 in the signature reveals a particularly nuanced immune disequilibrium: these three mechanistically distinct checkpoint axes converge to suppress cytotoxic T-cell responses and inflammatory resolution while paradoxically sustaining macrophage pro-inflammatory output through tryptophan metabolite accumulation and co-inhibitory receptor engagement on T cells (17, 18). It is important to note that the positive prognostic coefficients assigned to CD274 and LAG3 reflect their epidemiological co-elevation with adverse AMI outcomes in this transcriptomic dataset rather than a direct pro-inflammatory causal role. In the context of acute cardiac injury, upregulation of these checkpoint molecules most plausibly reflects reactive T-cell exhaustion and compensatory immunosuppression secondary to an overwhelming inflammatory insult—a state associated with impaired immune resolution and adverse remodeling. The co-occurrence of inflammasome hyperactivation and immune checkpoint exhaustion signatures in high-risk AMI, thus delineates a state of immune paralysis-in-activation, a pattern with expanding recognition in severe cardiovascular inflammatory states.

The marker-based immune scoring findings provide cellular context for the transcriptional patterns. The pronounced elevation of neutrophil and classical monocyte scores alongside the reciprocal reduction of NK-cell and CD8 T-cell scores in STEMI aligns mechanistically with the established pro-inflammatory myeloid surge following ischemia-reperfusion, during which early neutrophil infiltration and monocyte extravasation are essential for debris clearance but also drive maladaptive remodeling when excessively sustained (19). Prolonged myeloid predominance, captured by marker program scoring, has been independently linked to adverse ventricular remodeling and clinical heart failure in AMI populations (20). The concurrent transcriptomic suppression of cytotoxic lymphocyte programs may reflect T-cell exhaustion secondary to the overwhelming pro-inflammatory insult, a phenomenon that is increasingly recognized in severe cardiovascular inflammatory states (21).

Consensus clustering revealed two exploratory transcriptional subtypes among the 111 GSE59867 STEMI discovery samples. Subtype II (*n* = 62), characterized by higher neutrophil and classical monocyte scores and lower NK-cell and CD8 T-cell scores, showed a numerically higher proportion of 6-month heart failure events relative to Subtype I (*n* = 49), although the difference did not reach statistical significance in the 17 labeled samples available (Fisher's exact *p* = 0.153). These transcriptomic subtypes recapitulate the immune endophenotypes proposed in smaller single-institution studies (22) while extending their characterization to a multi-cohort transcriptomic context with greater statistical power. Subtype II patients represent the subgroup most likely to benefit from immune-modulatory therapeutic trials; currently evaluable candidates include tocilizumab (IL-6R blockade, with signals observed in ASSAIL-MI and ENTRACTE) (23), MCC950-class NLRP3 inhibitors currently advancing in phase II development, and pembrolizumab–relatlimab combination therapy exploiting dual PD-1/LAG-3 co-inhibitory blockade (24). IDO1 inhibitors, despite disappointing oncological trial results, may warrant re-evaluation in the distinct immunometabolic context of cardiac ischemia.

The cellular expression context from GSE121893 illustrated distinct cell-type profiles for the eight model genes, with BAX and EDA2R expressed across multiple lineages and MCEMP1 concentrated in macrophage populations. Because GSE121893 contains cardiac biopsy cells from patients with coronary disease, dilated cardiomyopathy, and normal hearts but no AMI specimens, these panels provide only cellular context and cannot validate AMI-specific gene localization. The complementary *in vitro* RT-qPCR results—demonstrating three- to fourfold upregulation of CXCL10, IL6, NLRP3, and CASP1 in ATCC-authenticated THP-1-derived macrophages stimulated with LPS/ATP and in EA.hy926 cells subjected to hypoxia/reoxygenation—confirm that these inflammatory transcripts are robustly induced under AMI-relevant stimulation conditions, providing mechanistic plausibility for their transcriptomic elevation in blood-based AMI datasets.

Several limitations of this investigation merit acknowledgment. The use of peripheral blood instead of infarct or peri-infarct myocardial tissue for discovery and validation requires caution when extrapolating blood-based signatures to intramyocardial inflammatory processes. The two exploratory binary-outcome analyses (6-month heart failure in 17 GSE59867 samples; 18-month recurrent events in 31 GSE48060 samples) were severely underpowered and relied on GEO-deposited metadata rather than individually adjudicated patient-level records, introducing potential outcome misclassification bias. No patient-level censoring times were available, precluding Kaplan–Meier or Cox regression analyses. The single-cell dataset (GSE121893) was derived from cardiac biopsy tissue of only eight individuals with coronary disease, dilated cardiomyopathy, or normal hearts and contained no AMI specimens; therefore, conclusions regarding AMI-specific cell-type gene localization cannot be drawn from this resource, and all related panels are presented as cellular context only. Cross-sectional transcriptomic datasets preclude the characterization of temporal immune dynamics across the acute, sub-acute, and chronic post-infarction phases. Prospective validation in a purpose-designed biomarker study—with protocol-standardized blood sampling, blinded centralized outcome adjudication, and mechanistic functional assays—is required before implementation in clinical decision-making. Although WGCNA was used solely for biological contextualization and did not participate in fold-wise feature selection, its module assignments were derived from the full-discovery dataset—incorporating information from later test-fold samples—representing a residual source of potential enrichment that prospective validation on an entirely independent cohort would resolve. Finally, while the inflammatory transcript findings generate compelling therapeutic hypotheses, none of the proposed agents has been formally tested in patients with AMI selected based on this molecular signature.

## Conclusions

By systematically applying nested machine learning to GEO transcriptomic data, we derived and validated an eight-gene diagnostic index (BAX, EDA2R, MCEMP1, SOCS3, ASGR2, RNASE1, ADM, and KPNA5) for acute myocardial infarction. The index achieved consistently high discriminative accuracy across three independent validation cohorts (AUCs: 0.863, 0.655, and 0.876) under leakage-controlled cross-validation. Marker-based immune scoring identified elevated myeloid (neutrophils and monocytes) and reduced lymphocyte (NK, CD8 T cells) programs as characteristic of STEMI, and *in vitro* RT-qPCR in ATCC-authenticated cell lines (THP-1 and EA.hy926) confirmed upregulation of inflammatory transcripts (CXCL10, IL6, NLRP3, and CASP1) under AMI-relevant stimulation conditions. Two exploratory binary-outcome analyses provided preliminary prognostic signals that require prospective validation with adequate sample sizes and patient-level follow-up data. Collectively, these data establish a leakage-controlled, multi-cohort-validated diagnostic gene index that constitutes a foundation for future prospective biomarker studies and the rational design of precision immune-targeted treatment strategies for AMI.

## Data Availability

The datasets presented in this study can be found in online repositories. The names of the repository/repositories and accession number(s) can be found in the article/Supplementary Material.

## Supplementary material

Supplementary Figure 1 Cross-cohort gene-direction and public outcome replacement for unsupported qPCR/survival panels. (**A–D**) Cohort-specific disease–reference mean log2 differences and 95% confidence intervals for BAX, EDA2R, MCEMP1, and SOCS3; fixed-effect summaries and I2 are shown descriptively. (**E**) Disease–reference differences for all eight model genes across five cohorts. (**F**) ROC curves for 6-month heart failure progression in GSE59867 and 18-month recurrent events in GSE48060. This supplementary figure contains only public data analyses; the *in vitro* RT-qPCR cell line experiment is shown in Figure 9.

## References

[1] Roth GA Mensah GA Johnson CO Addolorato G Ammirati E Baddour LM. Global burden of cardiovascular diseases and risk factors, 1990–2019. J Am Coll Cardiol. (2020) 76(25):2982–3021. 10.1016/j.jacc.2020.11.010 33309175 PMC7755038

[2] Thygesen K Alpert JS Jaffe AS Chaitman BR Bax JJ Morrow DA. Fourth universal definition of myocardial infarction. J Am Coll Cardiol. (2018) 72(18):2231–64. 10.1016/j.jacc.2018.08.1038 30153967

[3] Prabhu SD Frangogiannis NG. The biological basis for cardiac repair after myocardial infarction. Circ Res. (2016) 119(1):91–112. 10.1161/CIRCRESAHA.116.303577 27340270 PMC4922528

[4] Frangogiannis NG. The inflammatory response in myocardial injury, repair, and remodelling. Nat Rev Cardiol. (2014) 11(5):255–65. 10.1038/nrcardio.2014.28 24663091 PMC4407144

[5] Swirski FK Nahrendorf M. Cardioimmunology: the immune system in cardiac homeostasis and disease. Nat Rev Immunol. (2018) 18(12):733–44. 10.1038/s41577-018-0065-8 30228378

[6] Yan X Anzai A Katsumata Y Matsuhashi T Ito K Endo J. Temporal dynamics of cardiac immune cell accumulation following acute myocardial infarction. J Mol Cell Cardiol. (2013) 62:24–35. 10.1016/j.yjmcc.2013.04.023 23644221

[7] Lippi G Sanchis-Gomar F. Global epidemiology and future trends of heart failure. AME Med J. (2020) 5:15. 10.21037/amj.2020.03.03

[8] Ridker PM Everett BM Thuren T MacFadyen JG Chang WH Ballantyne C. Antiinflammatory therapy with canakinumab for atherosclerotic disease. N Engl J Med. (2017) 377(12):1119–31. 10.1056/NEJMoa1707914 28845751

[9] Abbate A Toldo S Marchetti C Kron J Van Tassell BW Dinarello CA. Interleukin-1 and the inflammasome as therapeutic targets in cardiovascular disease. Circ Res. (2020) 126(9):1260–80. 10.1161/CIRCRESAHA.120.315937 32324502 PMC8760628

[10] Langfelder P Horvath S. WGCNA: an R package for weighted correlation network analysis. BMC Bioinformatics. (2008) 9:559. 10.1186/1471-2105-9-559 19114008 PMC2631488

[11] Tibshirani R. Regression shrinkage and selection via the lasso. J R Stat Soc Series B. (1996) 58(1):267–88. 10.1111/j.2517-6161.1996.tb02080.x

[12] Song W. Current and emerging innovations in technology, strategy, and artificial intelligence: a new era in cardiac arrest and cardiopulmonary resuscitation. World J Emerg Med. (2025) 16(6):609–13. 10.5847/wjem.j.1920-8642.2025.120 41246533 PMC12616295

[13] Liu Q Zhang Y Sun J Wang K Wang Y Wang Y. Early identification of high-risk patients admitted to emergency departments using vital signs and machine learning. World J Emerg Med. (2025) 16(2):113–20. 10.5847/wjem.j.1920-8642.2025.031 40135217 PMC11930554

[14] Rosenberg S Elashoff MR Beineke P Daniels SE Wingrove JA Tingley WG. Multicenter validation of the diagnostic accuracy of a blood-based gene expression test for assessing obstructive coronary artery disease in nondiabetic patients. Ann Intern Med. (2010) 153(7):425–34. 10.7326/0003-4819-153-7-201010050-00005 20921541 PMC3786733

[15] Toldo S Abbate A. The NLRP3 inflammasome in acute myocardial infarction. Nat Rev Cardiol. (2018) 15(4):203–14. 10.1038/nrcardio.2017.161 29143812

[16] Nahrendorf M Swirski FK. Abandoning M1/M2 for a network model of macrophage function. Circ Res. (2016) 119(3):414–7. 10.1161/CIRCRESAHA.116.309194 27458196 PMC4965179

[17] Mellor AL Munn DH. IDO expression by dendritic cells: tolerance and tryptophan catabolism. Nat Rev Immunol. (2004) 4(10):762–74. 10.1038/nri1457 15459668

[18] Andrews LP Marciscano AE Drake CG Vignali DA. LAG3 (CD223) as a cancer immunotherapy target. Immunol Rev. (2017) 276(1):80–96. 10.1111/imr.12519 28258692 PMC5338468

[19] Epelman S Liu PP Mann DL. Role of innate and adaptive immune mechanisms in cardiac injury and repair. Nat Rev Immunol. (2015) 15(2):117–29. 10.1038/nri3800 25614321 PMC4669103

[20] Leuschner F Rauch PJ Ueno T Gorbatov R Marinelli B Lee WW. Rapid monocyte kinetics in acute myocardial infarction are sustained by extramedullary monocytopoiesis. J Exp Med. (2012) 209(1):123–37. 10.1084/jem.20111009 22213805 PMC3260875

[21] Vignali DA Collison LW Workman CJ. How regulatory T cells work. Nat Rev Immunol. (2008) 8(7):523–32. 10.1038/nri2343 18566595 PMC2665249

[22] Blanco P Palucka AK Pascual V Banchereau J. Dendritic cells and cytokines in human inflammatory and autoimmune diseases. Cytokine Growth Factor Rev. (2008) 19(1):41–52. 10.1016/j.cytogfr.2007.10.004 18258476 PMC2413068

[23] Kleveland O Kunszt G Bratlie M Ueland T Broch K Holte E. Effect of a single dose of the interleukin-6 receptor antagonist tocilizumab on inflammation and troponin T release in patients with non-ST-elevation myocardial infarction. Eur Heart J. (2016) 37(30):2406–13. 10.1093/eurheartj/ehw171 27161611

[24] Tawbi HA Schadendorf D Lipson EJ Ascierto PA Matamala L Castillo Gutiérrez E. Relatlimab and nivolumab versus nivolumab in untreated advanced melanoma. N Engl J Med. (2022) 386(1):24–34. 10.1056/NEJMoa2109970 34986285 PMC9844513

